# Case Report: A Novel Genetic Mutation Causes Idiopathic Infantile Arterial Calcification in Preterm Infants

**DOI:** 10.3389/fgene.2021.763916

**Published:** 2021-12-23

**Authors:** Liu Yunfeng, Han Tongyan, Wang Jing, Tong Xiaomei

**Affiliations:** Department of Pediatrics, Peking University Third Hospital, Beijing, China

**Keywords:** genetic mutation, case, preterm, infant, hypertensive, artery, calcification, report

## Abstract

Idiopathic infantile arterial calcification (IIAC), also known as generalized arterial calcification of infancy (GACI), is a heritable ectopic mineralization disorder that results in diffuse arterial calcifications and or stenosis, which are attributed to mutations in the ENPP1 gene. In this case study, we report the development of IIAC in a 2-month-old male preterm infant. The patient presented with severe hypertension and seizures, which revealed diffused calcifications and c.130C > T and c.1112A > T mutations in the ENPP1 gene. With biphosphonate, antihypertensive, and control epilepsy therapy, his blood pressure was maintained at 110–120/50–60 mmHg. Intellectual motor development retardation was anticipated in this patient. To the best of our knowledge, this is the first case in which a novel c.130C > T mutation in the ENPP1 gene has been identified, and the administration of bisphosphonates to patients with IIAC has been assessed.

## Introduction

Idiopathic infantile arterial calcification (IIAC), also known as generalized arterial calcification of infancy (GACI), is a rare autosomal recessive genetic disorder characterized by abnormalities in calcium and phosphorus metabolism. Approximately 69–80% of cases are caused by mutations in the ENPP1 gene on chromosome 6q23. A small fraction of cases results from mutations in the ABCC6 gene on chromosome 16p13.11. The first description of the clinical manifestation of GACI in the medical literature was published in 1901 (Bryant and White, 1901). IIAC is primarily diagnosed after observing hydroxyapatite deposits in the internal elastic layer of the large and medium arteries that causes vascular lesions, soft tissue calcification around joints, and various other life-threatening conditions due to the development of vascular lesions. Hypertension and heart failure are common clinical manifestations. Most infants develop arterial stenosis and heart failure within the first month of life. IIAC progresses rapidly; 80% of children with IIAC die within 6 months, and older children may die suddenly (Guimarães et al., 2010). This case report of neonatal GACI enriches the literature with data on a new familial mutation of the ENPP1 gene and provides details of the specific clinical course.

## Case Description

**FIGURE 1:**
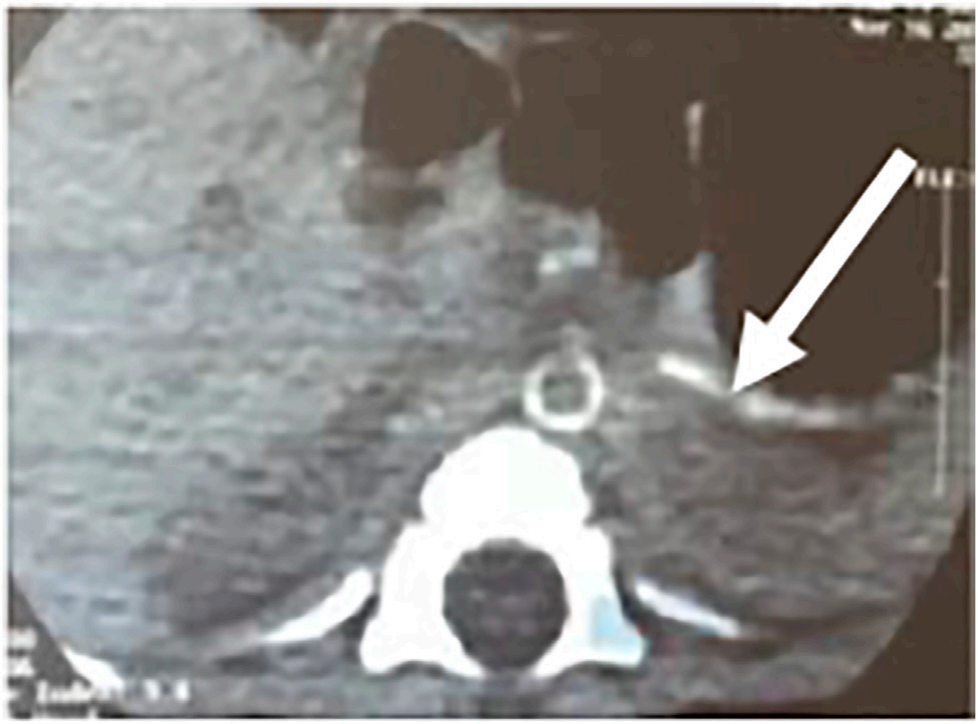
Enhanced contrast CT scan of the abdomen shows calcification of the abdominal aorta and its branches.

The boy was delivered at the 36th gestational period by cesarean section due to “suspected intrauterine distress” with a birth weight of 2,240 g. His Apgar scores were 7 at 1 min, 9 at 5 min, and 10 at 10 min. The amniotic fluid and umbilical cord were normal, and the placenta was calcified. The mother had two previous pregnancies and had complicated diabetes mellitus and hypothyroidism during pregnancy. At birth, he presented with respiratory distress, and blood gas analysis suggested severe respiratory failure. Echocardiography confirmed that the right ventricle was enlarged with moderate tricuspid regurgitation, and the pulmonary artery pressure was high, up to 70 mmHg. He developed severe hypertension 3 days after birth (systolic and diastolic blood pressure range, 100–120 and 40–70 mmHg, respectively). After Captopril treatment, the systolic blood pressure stabilized at 70–80/40–50 mmHg, and echocardiogram confirmed a thickened left ventricular wall, an enlarged left atrium, and a LVEF of 55%. Abdominal computed tomography (CT) detected diffuse calcifications of the abdominal aorta and its major branches and slight luminal stenosis (Figure 1).

**FIGURE 2:**
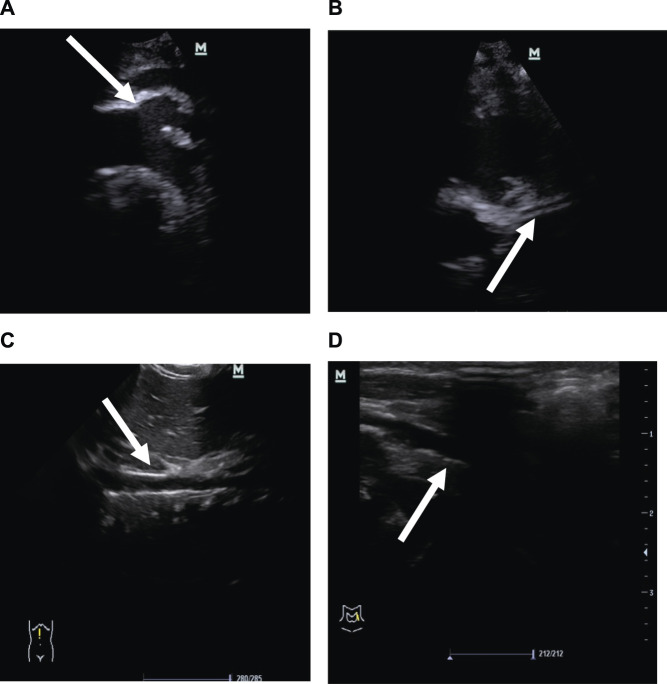
Vascular ultrasound images. **(A)** Strong echo in the aortic arch and its branches is indicated by the arrows. **(B)** Strong echo in the left coronary artery wall is indicated by the arrows. **(C)** Strong echo in the abdominal aortic wall is indicated by the arrows. **(D)** Strong echo in the femoral artery wall is indicated by the arrows.

**FIGURE 3:**
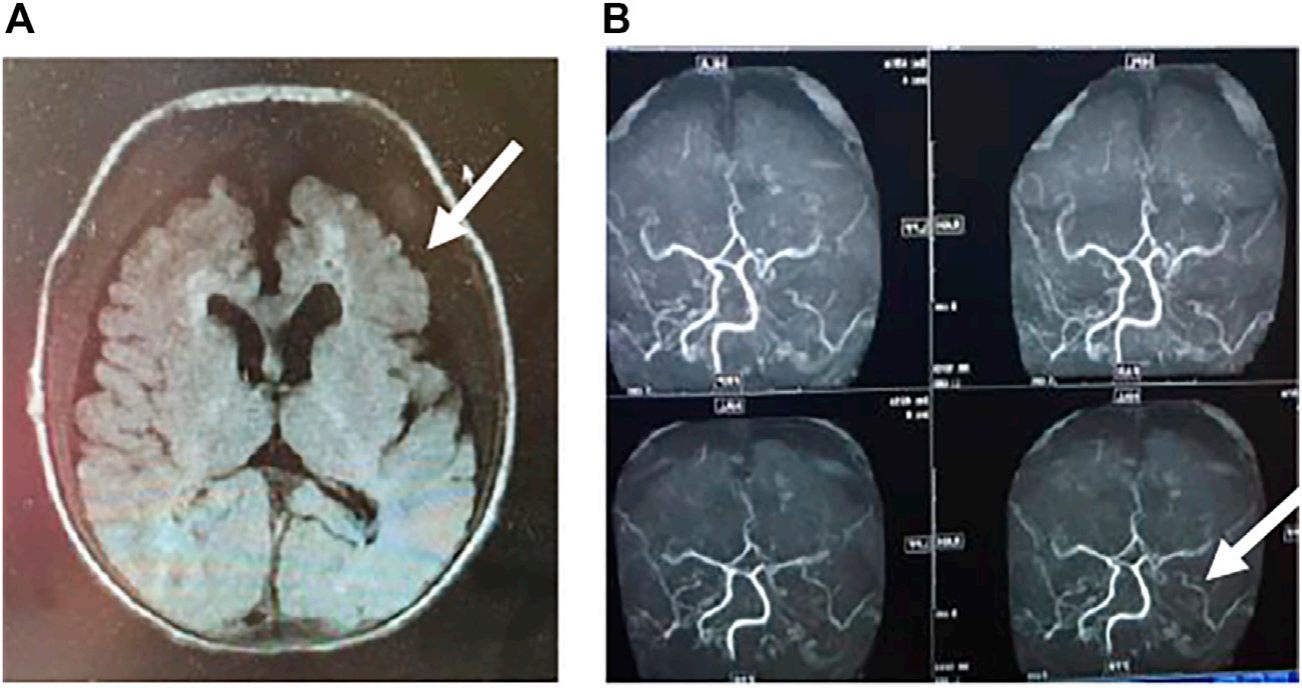
**A)** Cranial MRI showing bilateral frontal and temporal lobe atrophy and abnormally high signal in the white matter around the anterior horn of the ventricle. The lesions are indicated by arrows. **(B)** Cerebrovascular artery MRI showing multiple luminal stenoses of the bilateral anterior, middle, and posterior cerebral arteries and vertebral arteries. The lesions are indicated by arrows.

Two months after birth, he presented with a hypertensive crisis with severe heart failure and persistent convulsions. The liver and renal functions of the patient were abnormal. The systolic blood pressure was high, at 170 mmHg; the diastolic blood pressure was high, at 120 mmHg; the blood calcium level was low, at 1.6 mmol/L (normal value 2.11–2.52 mmol/L); and the blood phosphorus level was high, at 2.27 mmol/L (normal value 0.85–1.51 mmol/L). Chest radiography showed a few exudative lesions in both lungs and an enlarged cardiac shadow. Echocardiography revealed significant thickening of the left ventricular wall, enlarged left atrium and left ventricle, patent ductus arteriosus (small left-to-right shunt), and LVEF of 50%. The EEG had persistent multichannel sharp waves. Arterial Doppler ultrasound was performed and showed extensive calcification of the walls of the bilateral common carotid, bilateral femoral, carotid, vertebral, and internal carotid arteries, extensive calcification of the walls of the abdominal aorta and the right renal artery, and local luminal stenosis (Figures 2A–D). Flow velocity in the calcified abdominal aorta increased, and blood flow velocity in both renal arteries increased. Cranial MRI revealed bilateral frontal, parietal, and temporal lobe atrophy with dilated ventricles (Figure 3A). The cranial MRA showed bilateral internal carotid artery patchy luminal stenosis, left vertebral artery fibrillation, and left anterior cerebral artery, left middle cerebral artery luminal stenosis, and bilateral posterior cerebral artery luminal stenosis (Figure 3B). Cranial ultrasound showed bilateral calcification of the lenticular artery on both sides. No joint or skeletal calcification was observed on radiography, and prenatal ultrasound images were also retrospectively reviewed, but no arterial calcifications were detected.

**FIGURE 4:**
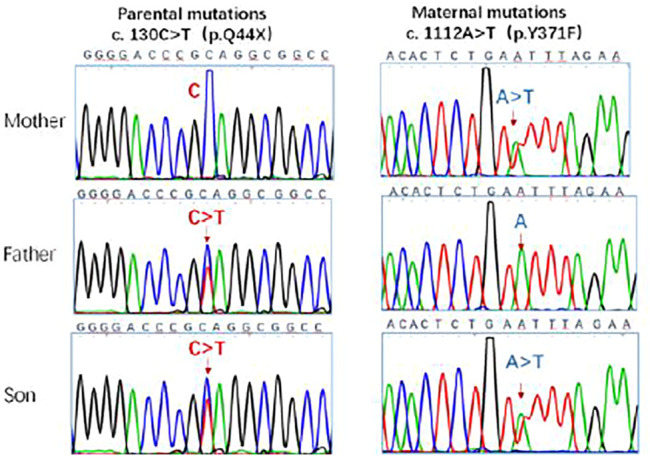
Sanger sequencing of ENPP1 in the parents and the patient. The c.130C > T, c.1112A > T locus mutations were detected in the gene. The ENPP1 gene was inherited from both parents with heterozygous c. 130 C > T and c. 1112A > T compound heterozygous mutations. The mutation sites are indicated by the arrows.

These singularities were consistent with those of the IIAC. The genotype of the patient was analyzed. Genetic testing of the child and parents suggested two heterozygous mutations in the ENPP1 gene *1*) c.130C > T (mutation of nucleotide 130 in the coding region from cytosine to thymine), resulting in an amino acid change p.Q44X (nonsense mutation). The father had a suspected heterozygous variant at this locus. *2*) c.1112A > T (mutation of nucleotide 1112 in the coding region from adenine to thymine), resulting in an amino acid change p.Y371F (mutation of amino acid 371 from tyrosine to phenylalanine) which is a missense mutation. The father showed no mutation at this locus, and the mother had a heterozygous variant at this locus. The genetic results were consistent with those for idiopathic infantile arterial calcification. The c.130C > T mutation has not been described in the literature or mutation databases (Figure 4).

The main treatment consisted of three courses: *1*) Circulatory system: Severe heart failure treatment with cardiac and vasodilator therapy with Foxglove preparation and sodium nitroprusside (0.1 µg/kg min to 4 µg/kg min) and oral administration of Norvasc and Losartan. The blood pressure of the patient gradually reduced to approximately 120/60 mmHg (95–99th percentile). *2*) Nervous system: The child developed a persistent state of convulsions and was considered to have hypertensive encephalopathy. Luminal and midazolam were administered successively, and continuous multichannel sharp wave discharges were observed in the EEG. After propofol was administered for 2 days, the abnormal EEG discharge was reduced, and propofol and midazolam dosages were gradually reduced. *3*) Zoledronic acid was administered intravenously (0.02 mg/kg once every other week for three times). Blood pressure was maintained at 110–130/60–70 mmHg (approximately 95th percentile of normal blood pressure in children of the same age). Blood calcium level was 1.92 mmol/L and blood phosphorus level was 2.68 mmol/L. His blood pressure stabilized, heart failure was corrected, no further convulsions occurred, and he was discharged in good condition.

During the long-time follow-up, with Norvasc and Losartan antihypertensive treatment, his blood pressure leveled at around 110–120/50–60 mmHg. Currently, he orally takes Etidronic acid as well as Clonazepam and Topamax to control epilepsy. His Gesell testing at 2 years old suggested he has moderate intellectual-motor retardation. At 3 years old, ultrasound showed that extensive vascular calcification was still present but was not progressing.The five-year-old child also suffers from cerebral palsy and receives rehabilitation training now.

His mother had another pregnancy 3 years ago, and prenatal genetic testing showed that the fetus carried the same mutations. A boy was born by full-term delivery, is now 3 years old, and is developing normally.

## Discussion

The first report of an association between IIAC and mutations in ENPP1 was reported by Rutsch et al. (2003). Animal experiments further confirmed that mutations in the ENPP1 gene can cause IIAC (Li et al., 2013, 2014). The gene is located on chromosomes 6q22 to q23, has a length of 83 kb, and contains 25 exons. ENPP1 is an external nucleotide pyrophosphatase/phosphodiesterase 1, an enzyme that causes vascular smooth muscle cells, chondrocytes, and osteoblasts to produce inorganic pyrophosphate (PPi). Mutations in the ENPP1 gene can lead to decreased levels of PPi and the production of hydroxyapatite crystals that are deposited in various layers of arteries, including the aorta, coronary arteries, heart valves, and renal arteries, causing vascular lesions, soft tissue calcification around joints, and various life-threatening conditions due to vascular lesions such as myocardial infarction, renal failure, cerebral infarction, and hypertensive encephalopathy (Numakura et al., 2006).

Less frequently, IIAC is linked to a mutation in the ATP-binding cassette subfamily C, member 6 (ABCC6) gene (GACI2, OMIM 614473) (Nitschke et al., 2012) which encodes ABCC6 (Nitschke and Rutsch, 2017). The ABCC6 gene on chromosome 16p13.11 is affected by the classic form of Pseudoxanthoma Elasticum (PXE). There is a genotypic overlap between IIAC and PXE, with some patients harboring mutations in ABCC6, and some patients with clinical manifestations consistent with PXE have mutations in ENPP1 (Nitschke et al., 2012). The main manifestations are calcification of the skin elastic fibers, systemic arterial vascular calcification, and cervical vertebral fusion (Ferreira et al., 2021a).

Genetic testing of our patient revealed two compound heterozygous mutations in the ENPP1 gene originating from his parents. Firstly, c.130C > T (mutation of nucleotide 130 in the coding region from cytosine to thymine) results in an amino acid change, p.Q44X, that causes a partial deletion of the protein from 44 amino acids onwards, a class I pathogenic form of mutation. This novel familial mutation in the ENPP1 gene has not yet been described in the literature or mutation databases. The other mutation is a missense mutation c.1112A > T (mutation of nucleotide 1112 in the coding region from adenine to thymine) that leads to an amino acid change, p.Y371F (mutation of amino acid 371 from tyrosine to phenylalanine), and is a pathogenic mutation that has been reported (Cheng et al., 2005) and is associated with IIAC.

The clinical manifestations of IIAC vary widely and can be broadly divided into two categories: early and late-onset (Sluis et al., 2006; Chong and Hutchins, 2008; Shaireen et al., 2013). Early onset (approximately 48%) refers to cases that occur in the fetal period or within 1 month of birth. The main manifestations are respiratory distress, fetal edema, severe heart failure, pulmonary hypertension, and hypertension. Late-onset (approximately 52%) refers to cases where the onset is 1 month after birth, and the main manifestations are respiratory failure, heart failure, cyanosis, hypertension, and nonspecific ischemic and hypoxic damage to other organs, such as abdominal pain and microvascular thrombosis (Rani et al., 2010; Zhang et al., 2011). Convulsive episodes and brain damage revealed old strokes and marked gliotic changes, but bilateral occipital necrosis is less commonly reported (Staretz-Chacham et al., 2019). If calcified plaques are deposited in the renal artery this can lead to renal artery stenosis and renal failure can occur. In our case, the child was born with heart failure, persistent hypertension, and progressive multisystem damage to liver function and kidney, consistent with the clinical features of the disease. The presence of the hypertensive crisis is suggestive of the aggressiveness of the disease. In contrast, the significant cerebrovascular calcification and neurological ischemic-hypoxic brain parenchymal damage presented in our case has not been reported previously.

Imaging examinations are of great value in the clinical diagnosis of IIAC. The main imaging manifestations are widespread calcification of the aorta and its branches, including the aortic arch, unnamed artery, abdominal aorta, femoral artery, and other large vessels. The inner wall of each vessel is not smooth; there are calcified plaques of different sizes attached, combined with stenosis of the diameter of the vessel, and the local blood flow velocity is significantly increased (Whitehall et al., 2003; Tran and Boechat, 2006; Bolster et al., 2015).

Based on the clinical features of large artery calcification combined with genetic testing, IIAC can be diagnosed (Borromini et al., 2016). Arterial biopsy indicates that hydroxyapatite deposits in the internal elastic membrane of large-and medium-sized arteries are the current gold standard bases for the diagnosis of IIAC (Dayapala et al., 2016).

The disease can be diagnosed during the fetal period. Fetal edema is the most common sign, and intrauterine distress and heart failure have been observed (Cansu et al., 2010; Esmer et al., 2015; Yi et al., 2017). Vascular ultrasound revealed intimal calcification and luminal stenosis of the aorta and abdominal aorta vessels, mostly in the second trimester. If an older sibling has a disease, the detection rate can be greatly improved by using multiple ultrasound examinations and genetic testing of amniotic fluid in the fetus of a subsequent pregnancy in early gestation (Mastrolia et al., 2015; Votava-Smith et al., 2017). In our case, no arterial calcifications were detected on prenatal ultrasound images.

As IIAC is a rare metabolic disorder, there are no guidelines for its treatment. Bisphosphonates are commonly used to treat IIAC, In 2008, (Rutsch et al., 2008). Rutsch used phosphonate analogs and performed a multicenter genetic study and retrospective observational analysis, suggesting that the survival of children was not significantly related to genetic mutations. Moreover, the survival rate was higher in children with well-controlled blood phosphate values and no hyperphosphaturia when treated with phosphonates. Therefore, phosphonate analogs are the first-line agents used in IIAC treatment (Ramjan et al., 2009). In recent years, different approaches for treating GACI have been intensively investigated, including early generation bisphosphonates, orally administered PPi, and soluble recombinant ENPP1-Fc protein (Albright et al., 2015; Dedinszki et al., 2017; Nitschke et al., 2018), and some cases have reported that sodium thiosulfate can improve ectopic calcification of IIAC (Omarjee et al., 2020), and animal experiments suggest that enzyme replacement therapy can have therapeutic effects on IIAC (Luo et al., 2020). The mechanism may be that this treatment reduces bone renewal, thus inhibiting further calcium deposition at sites of existing calcified injury, interferes with the formation of hydroxyapatite crystals, and/or provides analogs of inorganic pyrophosphates that can affect calcium deposition. Intravenous zoledronic acid therapy at doses of 0.25, 0.5, and 0.5 mg/kg for weeks 1, 2, and 3, respectively, and subsequent monthly doses of 0.5 mg/kg can be applied. It has recently been reported that switching administration to oral phosphonates at 20 mg/kg d after 3 weeks of intravenous therapy, with a gradual reduction to 50 mg/d for long-term oral maintenance, depending on vascular ultrasound treatment, can also provide relief (Edouard et al., 2011). In our case, treatment with symptomatic support and zoledronic acid every other week was switched to oral zoledronic acid after three courses, and blood pressure was largely maintained at the 95th–99th percentile of normal values in children of the same age, and the condition resolved. This also provides valuable insight for the treatment of other children with IIAC. The most common side effect of long-term administration of phosphonate preparations is severe skeletal lesions, similar to rickets manifestations (Otero et al., 2013; Ferreira et al., 2016) that must be monitored and treated.

The prognosis of patients with IIAC is poor. Combined coronary artery calcification is a major risk factor for a poor prognosis. Most deaths occur in infancy, and rapidly progressive malignant heart failure is often the main cause of death (Nael et al., 2014; Ferreira et al., 2021a). Individuals may die suddenly due to myocardial infarction. A small number of survivors may have intractable hypertension, recurrent cardiopulmonary failure (Nael et al., 2014) renal impairment, cervical spine fusion, and hearing loss (Ferreira et al., 2021b). Survivors require long-term oral antihypertensive drugs and medications to improve cardiac function. Patients with combined severe renal impairment require peritoneal dialysis (Stojanovic et al., 2013). Intractable heart failure eventually requires heart transplantation (Glatz et al., 2006; Giovannoni et al., 2014). Only two patients with IIAC who survived for 22 and 25 years have been reported. The patient in the present case successfully survived for 5 years, with hypertension and heart failure under effective control, but with severe brain damage and intellectual motor development retardation, and the long-term prognosis of the patient is not optimistic.

## Conclusion

IIAC is a rare autosomal recessive disorder characterized by abnormal calcium metabolism due to genetic mutations. A novel c.130C > T mutation in ENPP1 was identified. Hypertension, heart failure, and central nervous system involvement due to vascular calcification are typical clinical manifestations. Treatment with oral phosphonates is expected to improve the long-term prognosis. Early recognition of the disease by obstetricians enables intrauterine diagnosis and prenatal genetic screening, which can effectively improve informed healthcare decision-making for parents. The limitation of this case was that vascular calcification was not treated. In the follow-up, his vascular calcification did not deteriorate further.

## Data Availability

The original contributions presented in the study are included in the article/Supplementary Material, further inquiries can be directed to the corresponding authors.
